# Vertebral and Femoral Bone Mineral Density (BMD) Assessment with Dual-Energy CT versus DXA Scan in Postmenopausal Females

**DOI:** 10.3390/jimaging10050104

**Published:** 2024-04-27

**Authors:** Luca Pio Stoppino, Stefano Piscone, Sara Saccone, Saul Alberto Ciccarelli, Luca Marinelli, Paola Milillo, Crescenzio Gallo, Luca Macarini, Roberta Vinci

**Affiliations:** 1 Department of Medical & Surgical Sciences, Section of Diagnostic Imaging, University of Foggia, Viale Luigi Pinto n. 1, 71122 Foggia, Italy; stefano_piscone.549455@unifg.it (S.P.); sara.saccone@unifg.it (S.S.); saul_ciccarelli.520793@unifg.it (S.A.C.); luca.marinelli@unifg.it (L.M.); pmilillo@ospedaliriunitifoggia.it (P.M.); luca.macarini@unifg.it (L.M.); roberta.vinci@unifg.it (R.V.); 2 Department of Clinical and Experimental Medicine, University of Foggia, Viale Luigi Pinto n. 1, 71122 Foggia, Italy; crescenzio.gallo@unifg.it

**Keywords:** bone mineral density, dual-energy CT, DXA, osteoporosis, vertebral fractures, femoral neck fractures, dual-energy X-ray absorptiometry

## Abstract

This study aimed to demonstrate the potential role of dual-energy CT in assessing bone mineral density (BMD) using hydroxyapatite–fat material pairing in postmenopausal women. A retrospective study was conducted on 51 postmenopausal female patients who underwent DXA and DECT examinations for other clinical reasons. DECT images were acquired with spectral imaging using a 256-slice system. These images were processed and visualized using a HAP–fat material pair. Statistical analysis was performed using the Bland–Altman method to assess the agreement between DXA and DECT HAP–fat measurements. Mean BMD, vertebral, and femoral T-scores were obtained. For vertebral analysis, the Bland–Altman plot showed an inverse correlation (R^2^: −0.042; RMSE: 0.690) between T-scores and DECT HAP–fat values for measurements from L1 to L4, while a good linear correlation (R^2^: 0.341; RMSE: 0.589) was found for measurements at the femoral neck. In conclusion, we demonstrate the enhanced importance of BMD calculation through DECT, finding a statistically significant correlation only at the femoral neck where BMD results do not seem to be influenced by the overlap of the measurements on cortical and trabecular bone. This outcome could be beneficial in the future by reducing radiation exposure for patients already undergoing follow-up for chronic conditions.

## 1. Introduction

Low bone mineral density (l-BMD) and subsequent microarchitectural deterioration of bone texture leads to osteoporosis, a severe skeletal disorder resulting in increased bone fragility. This condition leads to an increased fracture risk even with minor trauma or during daily activities. The most commonly affected sites are the spine vertebrae, femur, humerus, wrist, and ankle bones. Due to osteoporosis not being a life-threatening condition, there is a scarcity of data from developing countries. Globally, osteoporosis leads to over 8.9 million fractures each year, equating to an osteoporotic fracture occurring every 3 s [1]. Osteoporosis is estimated to impact 200 million women globally, with 27 million individuals affected solely in Europe [2,3,4].

In the literature, two types of osteoporosis have been described: primary and secondary type. There are two subtypes of primary osteoporosis, type I or postmenopausal and type II or senile; by contrast, secondary osteoporosis might be caused by other health conditions or medical treatment. Osteoporosis affects 10.2% of adults aged 50 and above, and its incidence is expected to rise to 13.6% by 2030. Osteoporotic fractures significantly affect morbidity, mortality, and quality of life, so screening activities should include all women over 65 and postmenopausal women with any clinical risk factor [5].

Due to its increased incidence over the last 40 years, several novel techniques and technologies have been developed to better detect it. Dual-energy X-ray absorptiometry (DXA) is used to determine an individual’s bone mineral density by combining two X-ray beams with different energy levels. From 1994 onwards, two parameters have been established worldwide to assess osteoporosis, known as the “T-score” and “Z-score”.

The “T-score” is a statistical value representing the deviation of the subject’s bone mineral density from the reference population (healthy population of the same sex aged 20–30 years) in terms of standard deviations. According to the World Health Organization (WHO), osteoporosis is diagnosed when bone mineral density is equal to or less than −2.5 standard deviations below that of a young, healthy adult female reference population, so when the T-score is equal to or below −2.5. A T-score between −1 and −2.5 indicates osteopenia.

The “Z score”, on the other hand, indicates how many standard deviations the bone mineral density of a patient differs from that of a healthy population of the same age and gender [6,7,8].

Although DXA is considered the gold standard, some studies have raised concerns about its importance in early osteoporosis detection [9]. DXA is projection-dependent and cannot differentiate trabecular from cortical BMD, and it can also be influenced by adjacent vessel calcifications, tissue thickness, intestinal content, and degenerative bone alterations [10].

In recent years, numerous technical advances in the field of dual-energy computed tomography (DECT) have expanded its utilization within routine clinical practice [11,12,13]. Material-specific attenuations observed in low- and high-energy spectra enable the relatively precise characterization of the material composition within a given object [14,15]. The technique is most commonly employed in the musculoskeletal system for the assessment of uric acid crystal depositions in gout arthropathy [12].

Moreover, the acquisition of virtual monochromatic images (VMIs) offers several advantages in visualizing iodine contrast and reducing metal implant artifacts [16], utilizing the energy-dependent photoelectric effect across different X-ray spectra. Indeed, virtual monoenergetic imaging (VMI) allows for the calculation of image data of different energy levels from a single CT scan.

DECT has provided valuable clinical information for musculoskeletal applications and bone mineral density (BMD) assessment with minimal influence from marrow edema [17].

Previous studies with DECT have assessed the potential use of specific base material pairs to detect changes in BMD. Li et al. demonstrated that they could achieve high accuracy in BMD provided there was a smaller deviation in DECT when using hydroxyapatite and water as the base material pair [18]. Yue et al. reported that using calcium and water as base materials could reflect the age-related changes in the lumbar spine of adult women and its correlation with BMD [19]. Wang et al. proved that measurement using the hydroxyapatite–water base material pair provides high diagnostic accuracy [20].

Our study was performed on the base material pairing of two of the bone’s major components: hydroxyapatite and fat. The aim of this study is to demonstrate the potential use of dual-energy CT using a HAP–fat material pairing for osteoporosis evaluation in postmenopausal women. This approach aims to provide a diagnostic tool by combining osteoporosis screening during routine CT exams.

## 2. Materials and Methods

### 2.1. Study Population

This retrospective opportunistic study included 51 postmenopausal female patients who underwent both DXA and dual-energy CT of the lumbar spine, abdomen, and pelvis in 2022–2023 for oncological follow-up or other clinical evaluation. According to the WHO, the study population was divided into three groups: osteoporotic (T-score is equal or less than −2.5), osteopenic (T-score between −1 and −2.5), and healthy patients (T-score more than −1).

Only patients with a maximum time gap of 6 months between DXA and DECT exams were included. Patients with focal bone lesions, clinical or radiological disease, prosthetic materials, postsurgical outcomes, or prior fractures were excluded from the study (Table 1).

### 2.2. Imaging

All images were acquired using a 256-slice multidetector CT system with spectral imaging capability (Revolution, GE Healthcare, Chicago, IL, USA) using a 1.0 mm slice thickness and tube current of 200 mA (Dose Right automatic exposure control system). Conventional 80 and 140 kVp images, as well as material maps, were primarily reconstructed with a thickness of 0.625 mm in a medium soft-tissue kernel and subjected to further postprocessing.

The CT data were reconstructed with Gemstone Spectral Imaging (GSI) data, and MPR reconstructions were performed in coronal and sagittal planes. Virtual monochromatic images were generated from the portal venous phase, which was acquired using rapid kVp-switching DECT (80/140 kVp) with GSI preset at a pitch of 0.984 and rotation time of 0.8 s. A bodyweight-adapted injection (1.4 mL/kg) of iohexol 350 mgI/mL (Omnipaque 350, GE Healthcare) was performed at a rate of 3.5 mL/s. As demonstrated by Ulas et al., we used an energy level of 140 kVp since it provides a better SNR compared to the other monochromatic reconstruction [21]. In order to reduce artifacts from arteriovenous opacification, we avoided measuring the areas adjacent to the vessels. In addition, bone segments that revealed artifacts were not included in the study.

**Figure 1:**
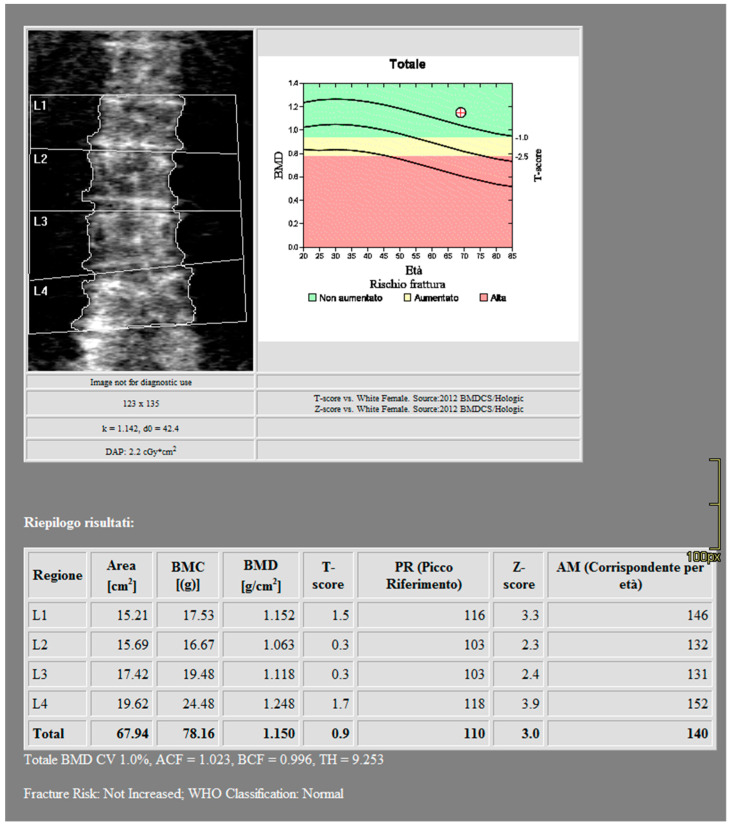
DXA measurements on the lumbar vertebrae (Regione: measurement region; Area: measurement area; BMC: Bone Mineral Content; BMD: Bone Mineral Density; PR: Reference peak; AM: Age Matched).

**Figure 2:**
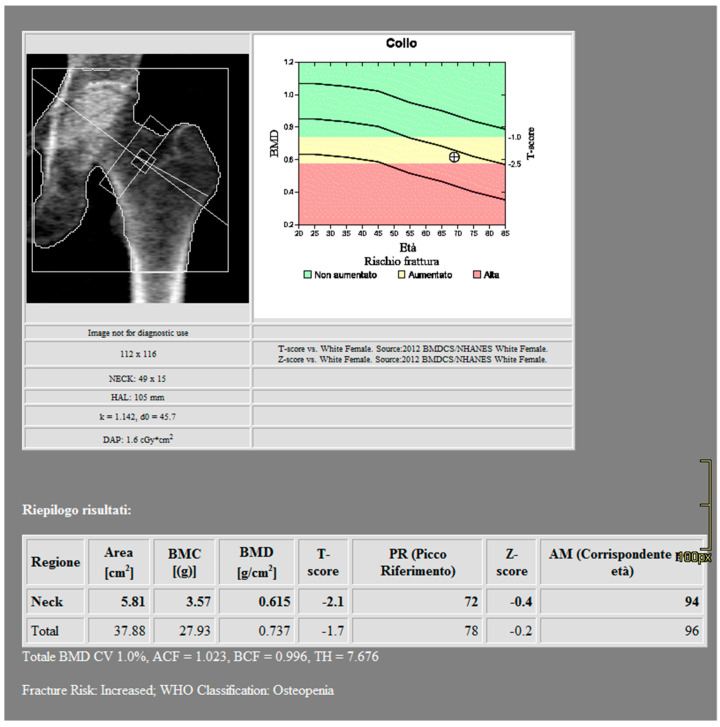
DXA measurements on the femoral neck (Regione: measurement region; Area: measurement area; BMC: Bone Mineral Content; BMD: Bone Mineral Density; PR: Reference peak; AM: Age Matched).

DXA scans were performed using a bone densitometer (Discovery A, HOLOGIC, Marlboroug, MA, USA) for the lumbar vertebrae (L1 to L4) and femoral neck (Figure 1 and Figure 2).

### 2.3. Postprocessing

**Figure 3:**
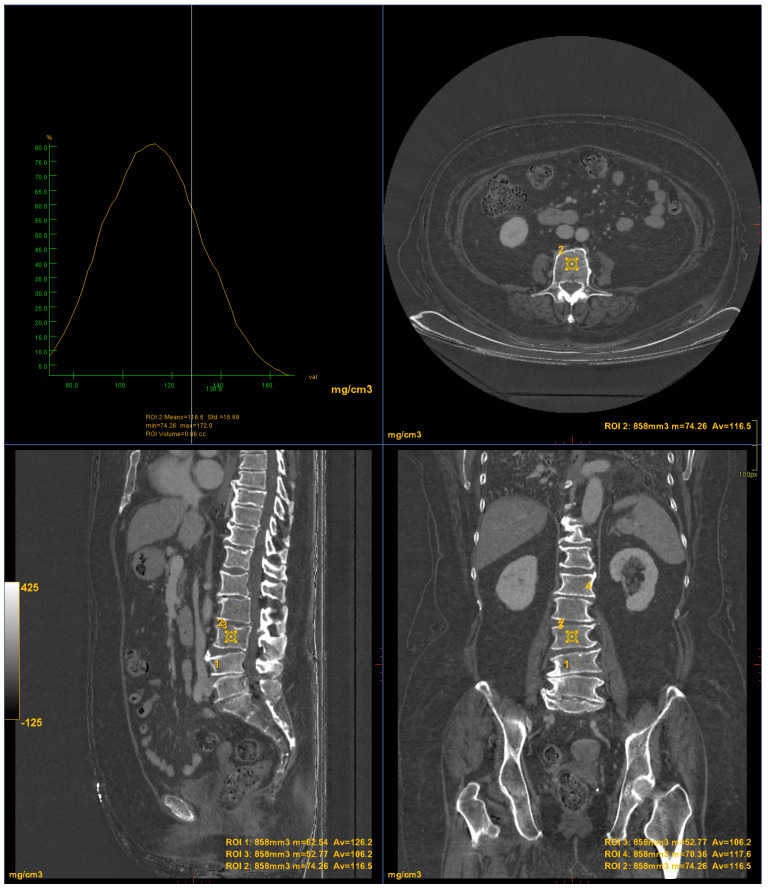
DECT measurements on the lumbar vertebrae (ROI1: L4 measurement; ROI2: L3 measurement; ROI3: L2 measurement; ROI4: L1 measurement). The ROI volume was 858 mm^3^.

**Figure 4:**
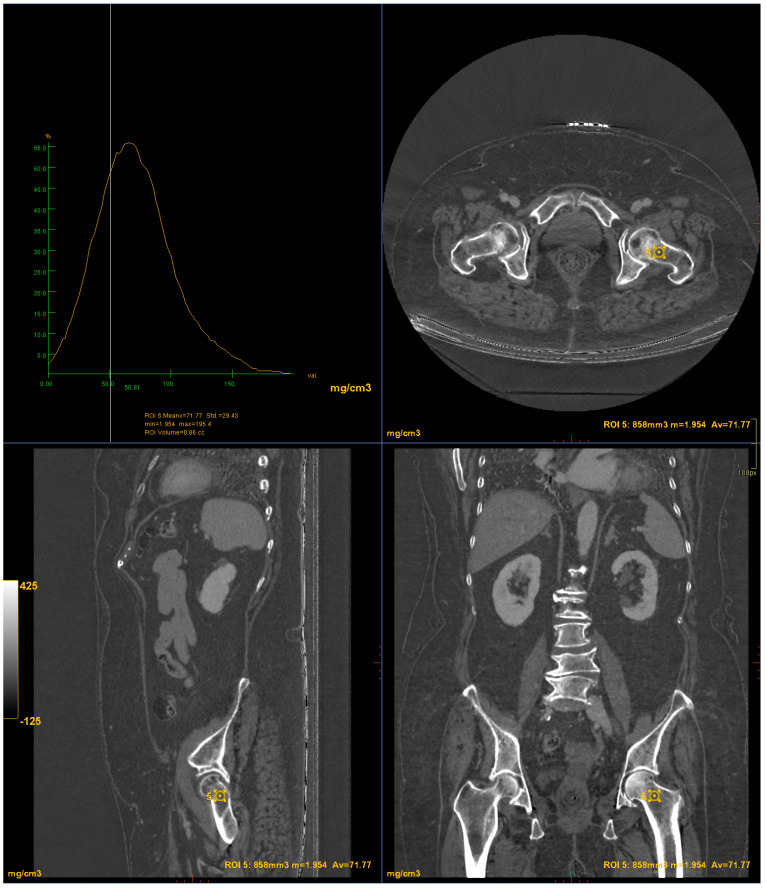
DECT measurements on the femoral neck (ROI5: femoral neck volume measurement). The ROI volume was 858 mm^3^.

CT images were processed using AW3.2 software (GE Healthcare, Chicago, IL, USA) with a bone window and HAP–fat base material pairing, which highlights structures containing hydroxyapatite. Three-dimensional volume of interest (VOI) measurements were obtained at the lumbar vertebrae (Figure 3) and femoral neck (Figure 4), sampling the trabecular bone while excluding cortical bone regions.

### 2.4. Data Evaluation and Statistical Analysis

Based on the T-score values obtained from DXA (the reference standard), the performance results were reported in terms of accuracy (proportion of correct prediction), confusion matrix, sensitivity (proportion of correctly identified actual positives), and specificity (proportion of correctly identified actual negatives). Using a BMD cutoff from DXA, true positive (osteoporotic, osteopenic) was defined when the T-score was less than or equal to −1, while true negative was defined when the T-score was >−1.

Based on the data obtained from DECT, we divided the study population into three risk classes: class risk 0 when HAP–fat values were >165 mg/cm^3^, risk class 1 when values were between 135 and 165 mg/cm^3^, and risk class 2 when values were <135 mg/cm^3^. Similarly, DXA values were also divided into three risk classes: class risk 0 for healthy patients, class risk 1 for the osteopenic group, and class risk 2 for the osteoporotic group.

All statistical analyses were performed using Jamovi software (The Jamovi Project 2024; *jamovi* Version 2.5 [computer software]; retrieved from https://www.jamovi.org). Continuous data with a normal distribution are presented as mean and standard deviations (mean ± SD). The Kruskal–Wallis one-way analysis of variance (ANOVA) test was performed to compare absolute mean values of DXA and DECT HAP–fat obtained in the three groups of patients (osteoporosis, osteopenia, and normal) for both lumbar vertebrae and femoral neck. The assumption of normal distribution was verified using the Shapiro–Wilk test. Post hoc analysis was performed using Dwass–Steel–Critchlow–Fligner pairwise comparisons, when appropriate. The Bland–Altman method was used to evaluate the agreement between DXA and DECT HAP–fat measurements, using the same three risk classes for both methods. In addition, the coefficient of determination (R^2^) and residual root-mean-square error (RMSE) were calculated to evaluate the goodness of the linear fits.

## 3. Results

The baseline characteristics of the 51 patients participating in the study are presented in Table 2 and Table 3. According to the BMD measurement on DXA, 15 participants were diagnosed in the osteoporotic range in the lumbar area and 15 participants in the hip area. Additionally, 27 participants were identified in the osteopenic range in the lumbar area and 24 participants in the hip area. Nine subjects were classified in the normal range in the lumbar area and twelve subjects in the hip area. 

The HAP–fat pairing of base materials for the evaluation of BMD in the lumbar vertebrae showed a diagnostic accuracy of 67%, sensitivity of 76%, and specificity of 22%, while for the evaluation of BMD in the femoral neck, a diagnostic accuracy of 82%, sensitivity of 77% and a specificity of 100% were achieved.

**Figure 5:**
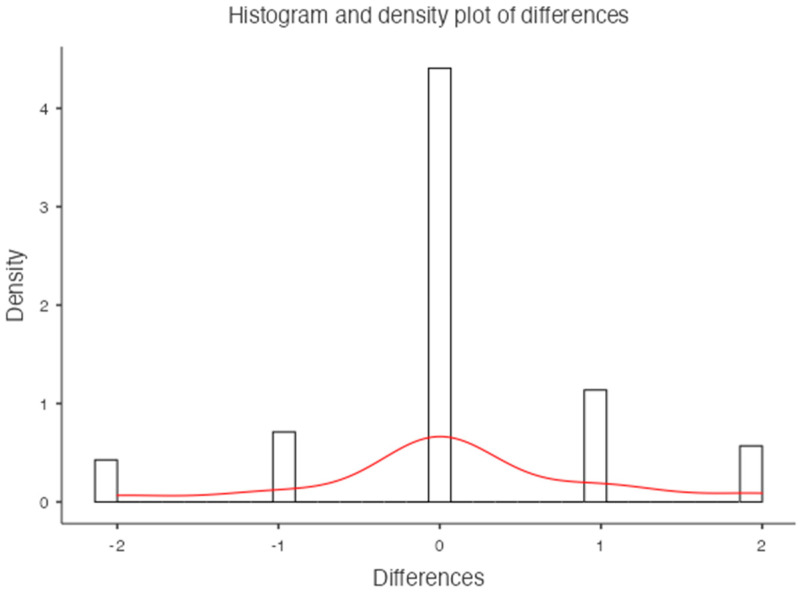
Histogram and density plot of differences for the lumbar vertebrae.

**Figure 6:**
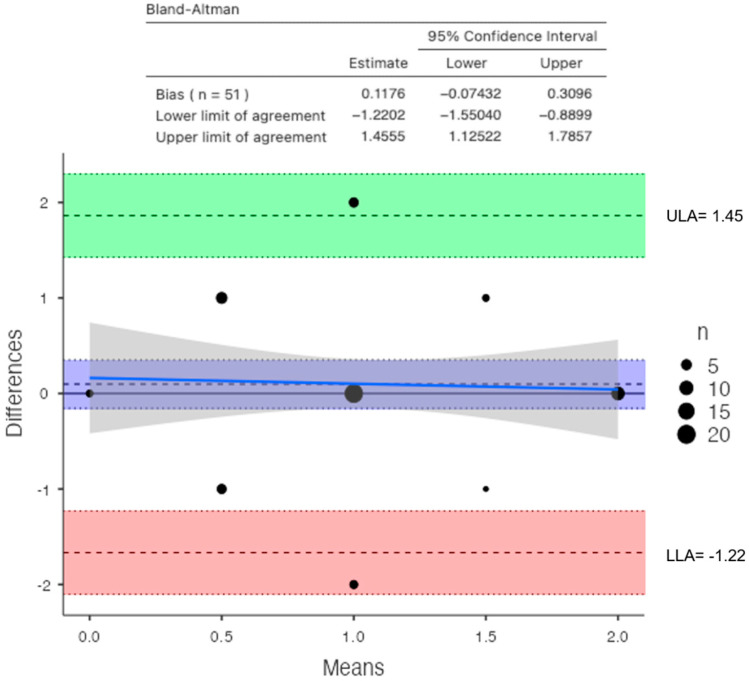
Bland–Altman plot between DXA and DECT HAP–fat measurements on the lumbar vertebrae (direction of subtraction for the Y axis—DXA minus DECT; blue line shows the performance bias of the method).

For the lumbar vertebrae, the mean BMD and T-score on DXA images were 0.648 g/cm^2^ and −3.66 for the osteoporosis group, 0.855 g/cm^2^ and −1.73 for the osteopenic group, and 1.02 g/cm^2^ and −0.83 for the normal group, respectively (Figure 5). There was a significant difference among the osteoporosis, osteopenic, and normal groups in BMD and T-score (all *p* < 0.001). The mean HAP value on DECT images was 144.86 mg/cm^3^ for the osteoporosis group, 162.49 mg/cm^3^ for the osteopenic group, and 139.57 mg/cm^3^ for the normal group (*p* = 0.2591) (Table 2). Bland–Altman analysis revealed a bias of 0.09, with 95% limits of agreement from −1.6 to 1.8 (S.D. ± 0.9001; *p* = 0.4) (Figure 6). 

**Figure 7:**
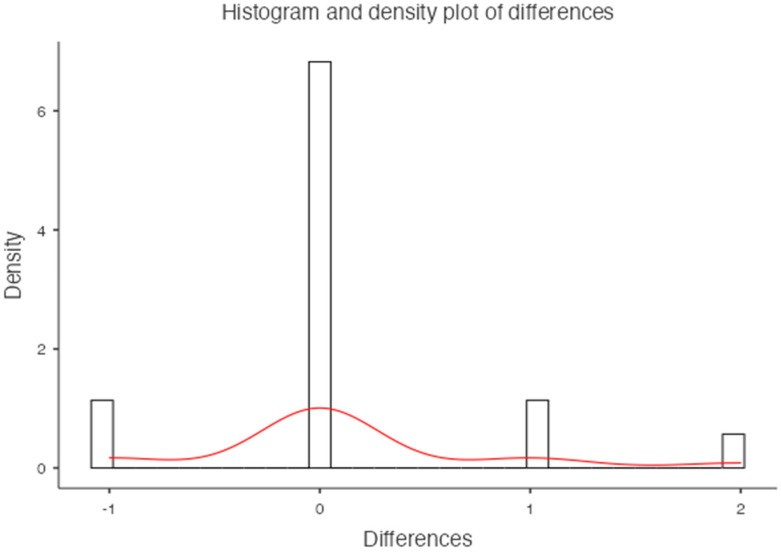
Histogram and density plot of differences for the femoral neck.

**Figure 8:**
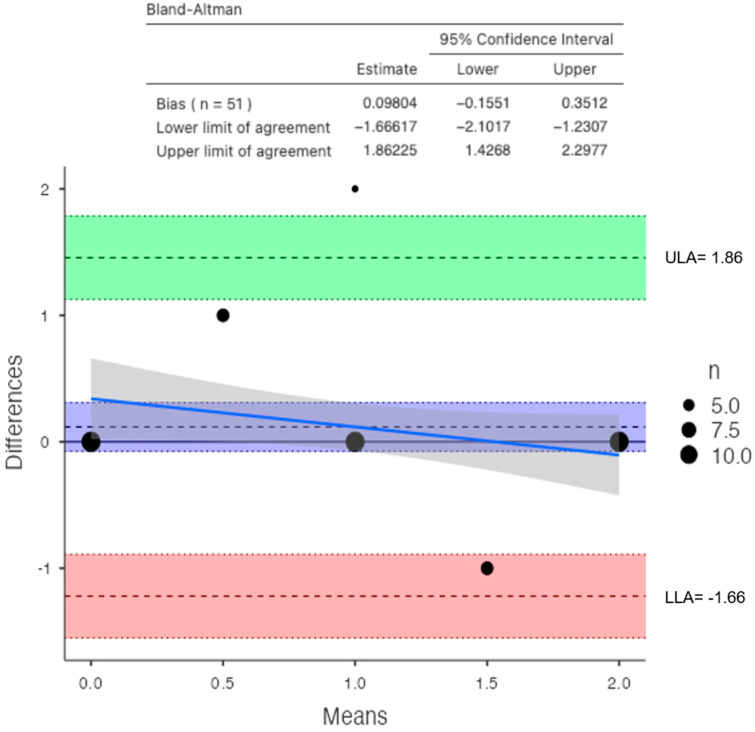
Bland–Altman plot between DXA and DECT HAP–fat measurements on the femoral neck (direction of subtraction for the Y axis—DXA minus DECT; blue line shows the performance bias of the method; ULA: Upper Limit of Agreement; LLA Lower Limit of Agreement).

For the femoral neck, the mean BMD and T-score on DXA images were 0.522 g/cm^2^ and −2.92 for the osteoporosis group, 1.19 g/cm^2^ and −1.7 for the osteopenic group, and 1.98 g/cm^2^ and −0.68 for the normal group, respectively (Figure 7). There was a significant difference among the osteoporosis, osteopenic, and normal groups in BMD and T-score (all *p* < 0.001). The mean HAP value on DECT images was 74.71 mg/cm^3^ for the osteoporosis group, 107.57 mg/cm^3^ for the osteopenic group, and 121.93 mg/cm^3^ for the normal group (*p* < 0.001) (Table 3). Bland–Altman analysis revealed a bias of 0.11, with 95% limits of agreement from −1.2 to 1.4 (S.D. ± 0.6826; *p* = 0.2) (Figure 8).

An inverse correlation was observed between T-score and DECT HAP–fat measurements on the lumbar vertebrae (R^2^: −0.042; RMSE: 0.690). In contrast, a good linear relationship was found between T-score and DECT HAP–fat measurements on the femoral neck (R^2^: 0.341; RMSE: 0.589). 

The Kruskal–Wallis one-way analysis of variance (ANOVA) test for the analysis of the dataset obtained on the lumbar vertebrae did not show statistical significance (*p* = 0.063). Post hoc analysis with Dwass–Steel–Critchlow–Fligner pairwise in femoral data comparisons showed significant differences between osteoporosis and healthy groups (*p* < 0.001) and osteopenic and normal groups (*p* < 0.001), while differences between osteoporosis and osteopenic groups were not found to be significant.

## 4. Discussion

Although DXA is still considered to be the gold standard in osteoporosis diagnostic programs, several studies have reported the opportunistic role of CT scan as a useful tool for assessing BMD [22] since DXA cannot be used in patients with scoliosis or calcifications from chronic disease, metal implants in both hips or at multiple levels of the spine, or cement in a vertebral body. In addition, DXA cannot distinguish cancellous bone from cortical bone quality as it is based on two dimensions. As shown by Pickhardt and colleagues [23], the use of CT-derived lumbar BMD vs. DXA measurements is helpful. In a population study of almost 2000 adults, CT scans were both specific and sensitive (>90%) in osteoporosis detection compared to DXA scans.

DXA may introduce variability in measurements due to overlying soft tissues, vascular calcifications, intestinal contents, and degenerative bone changes, potentially influencing results. Similar to other studies in the literature, we chose to focus exclusively on the evaluation of the trabecular component to avoid the potential for a false increase in BMD values due to the causes mentioned above [24,25]. Additionally, trabecular bone is more susceptible to involution in osteopenic or osteoporotic processes.

In this retrospective study, we demonstrated the potential of DECT in obtaining BMD values in mg/cm^3^ using a specific base material pairing [26]. In fact, the use of the HAP–fat pair allowed for obtaining additional information in routine scans without further radiation exposure. In a single scan, DECT has the capability to achieve material separation and generate various base material pairs based on clinical requirements, accompanied by a set of monochromatic images. VMI stands as one of the intrinsic reconstruction techniques in DECT. Adapted to specific requirements, its main purpose is to optimize imaging for routine diagnostics [27,28]. Using lower-energy-level VMI results in enhanced contrast but also increased image noise, leading to a lower signal-to-noise ratio (SNR). Higher energy levels (140 kVp) did not result in an improved SNR [17].

Some experimental and clinical studies indicate that the density value of the base substance can effectively represent the material content when the chosen base material pairs closely match the actual material. Similar to the findings of Deng et al., we found that the HAP concentration using the HAP–fat material pair was correlated with BMD, and the CT value (HU) was also measured on the DECT monochromatic images that are less susceptible to beam hardening artifacts [24].

In contrast to Gruenewald et al. [25], our analysis not only assessed the lumbar vertebrae (L1 to L4) but also extended to include the femoral neck. Unlike other studies, we did not find a statistically significant correlation between vertebral values obtained with DXA and DECT, as DECT assesses the true volumetric BMD of trabecular bone, while DXA measures it in reference to a surface, including both cortical and trabecular components [29,30]. In fact, our study demonstrated low specificity for the assessment of vertebral BMD. By contrast, we found a significant correlation between femoral measurements from DXA and DECT, which showed high accuracy, sensitivity, and specificity in BMD evaluation. As other studies anticipated, there are many vertebral and paravertebral alterations that contribute to the evaluation of BMD [29,30], justifying the lack of correlation of data between DXA and DECT. This is further confirmed by the association between DXA results and the data on the femoral neck since there are fewer alterations that can invalidate the assessments performed with DXA. Specific BMD values obtained with DECT for osteoporosis diagnosis should be further developed in future studies with larger patient cohorts, making DECT a routine technique for osteoporosis diagnosis. Moreover, patients with chronic conditions undergoing DXA for BMD evaluation and additional DECT for disease follow-up could benefit from a BMD calculation through routine DECT, minimizing radiation exposure [31,32].

This study has several limitations. The first is that the literature lacked DECT reference values for BMD. The second is that iodine contrast media were administered in all patients since this is an opportunistic study performed during the follow-up of other pathologies. Therefore, bone marrow enhancement could represent a source of error in VOI measurements. Moreover, this study did not assess the risk factors for BMD alterations, as patients were opportunistically studied during routine exams for other purposes, and external factors (medications, comorbidities, etc.) were not considered. The fourth is that the HAP–fat algorithm was vendor-specific, making our results non-reproducible with DECT from different vendors. Furthermore, only postmenopausal women were included in this study, while WHO T-score standards encompass both men and premenopausal women. The last limitation is that data collection involved four different operators for different patients, potentially introducing variability in VOI positioning. An automatic trabecular bone density calculation algorithm would reduce this variability.

## 5. Conclusions

In conclusion, a significant correlation was observed between the T-score and DECT HAP–fat measurements in the femoral neck. Unlike the lumbar vertebrae, the femoral neck represents a skeletal site less influenced by the overlap of the measurements on cortical and trabecular bone. Therefore, DECT scans performed on the femoral neck may be used to accurately assess BMD and fracture risk in postmenopausal women, improving the ability to monitor disease progression and providing a more accurate assessment. This outcome could be beneficial in the future by reducing radiation exposure for patients already undergoing follow-up for chronic conditions.

## Figures and Tables

**Table 1:** Inclusion and exclusion criteria.

Inclusion Criteria	Exclusion Criteria
Postmenopausal women	History of prior fractures or focal bone lesions
Patients undergoing oncologic follow-up	Presence of prosthetic materials
Maximum gap of 6 months between DXA and DECT	Postsurgical patient

**Table 2:** Characterization of the study population for lumbar vertebrae analysis.

Characteristics	Osteoporosis	Osteopenia	Normal	*p*-Value
Mean age (range)	66.4 ± 5.91 (58–73)	63.5 ± 4.89 (47–70)	68 ± 1.22 (65–70)	
Patients	15	27	9	
T-score	−3.66 ± 1.79	−1.73 ± 0.34	−0.83 ± 0.11	<0.001
BMD (g/cm^2^)	0.648 ± 0.07	0.855 ± 0.04	1.02 ± 0.11	<0.001
HAP–fat (mg/cm^3^)	144.86 ± 34.7	162.49 ± 12.66	139.57 ± 24.25	0.2591

**Table 3:** Characterization of the study population for femoral neck analysis.

Characteristics	Osteoporosis	Osteopenia	Normal	*p*-Value
Mean age (range)	66.4 ± 5.91 (58–73)	63.5 ± 4.89 (47–70)	68 ± 1.22 (65–70)	
Patients	15	24	12	
T-score	−2.92 ± 1.13	−1.7 ± 0.43	−0.68 ± 0.22	<0.001
BMD (g/cm^2^)	0.522 ± 0.04	1.19 ± 0.09	1.98 ± 2.21	<0.001
HAP–fat (mg/cm^3^)	74.71 ± 17.7	107.57 ± 7.84	121.93 ± 14.43	<0.001

## Data Availability

The data presented in this study are available upon request from the corresponding author.
